# Telemonitoring in diabetes: evolution of concepts and technologies, with a focus on results of the more recent studies

**DOI:** 10.25122/jml-2019-0006

**Published:** 2019

**Authors:** Emmanuel Andrès, Laurent Meyer, Abrar-Ahmad Zulfiqar, Mohamed Hajjam, Samy Talha, Thibault Bahougne, Sylvie Ervé, Jawad Hajjam, Jean Doucet, Nathalie Jeandidier, Amir Hajjam El Hassani

**Affiliations:** 1. Service de Médecine Interne, Diabète et Maladies Métaboliques de la Clinique Médicale B, Hôpitaux Universitaires de Strasbourg, 1, porte de l’Hôpital, 67091 Strasbourg cedex France; 2. Equipe de recherche EA 3072 «Mitochondrie, Stress oxydant et Protection musculaire», Faculté de Médecine de Strasbourg, Université de Strasbourg (Unistra), Strasbourg, France; 3. Service d’Endocrinologie et de Diabétologie de la Clinique Médicale B, Hôpitaux Universitaires de Strasbourg, Strasbourg, France; 4. Service de Médecine Interne, Gériatrie et Thérapeutique, CHU de Rouen, France; 5. Predimed Technology, Strasbourg, France; 6. Service de Physiologie et d’Explorations Fonctionnelles, Hôpitaux Universitaires de Strasbourg, Strasbourg, France; 7. Centre d’expertise des Technologies de l’Information et de la Communication pour l’autonomie (CENTICH) et Mutualité Française Anjou-Mayenne (MFAM), Angers, France; 8. Equipe de recherche EA 4662 «Nanomédecine, Imagerie, Thérapeutiques», Université de Technologie de Belfort-Montbéliard (UTBM), Belfort-Montbéliard, France

**Keywords:** artificial intelligence, chronic disease, diabetes, information and communication technology, Internet, telemedicine, telemonitoring, Web

## Abstract

This is a narrative review of telemonitoring (remote monitoring) projects and studies within the field of diabetes, with a focus on results of the more recent studies. Since the beginning of the 1990s, several telemedicine projects and studies focused on type 1 and type 2 diabetes. Over the last 5 years, numerous telemedicine projects based on connected objects and new information and communication technologies (ICT) (elements defining telemedicine 2.0) have emerged or are still under development. Two examples are the DIABETe and Telesage telemonitoring project which perfectly fits within the telemedicine 2.0 framework – the first to include artificial intelligence (AI) with MyPredi^TM^ and Diabeo^TM^. Mainly, these projects and studies show that telemonitoring diabetic result in: improvements in control of blood glucose (BG) level and significant reduction in HbA1c (e.g., for Telescot et TELESAGE studies); positive impact on co-morbidities (arterial hypertension, weight, dyslipidemia) (e.g., for Telescot and DIABETe studies); better patient’s quality of life (e.g., for DIABETe study); positive impact on appropriation of the disease by patients and/or greater adherence to therapeutic and hygiene-dietary measures (e.g., The Utah Remote Monitoring Project); and at least, good receptiveness by patients and their empowerment. To date, the magnitude of its effects remains debatable, especially with the variation in patients’ characteristics (e.g., background, ability for self-management, medical condition), samples selection and approach for the treatment of control groups. All of the recent studies have been classified as “Moderate” to “High”.

## Introduction

Intensive glucose control has been shown to delay or prevent the development of micro- and macro-vascular complications related to diabetes. However, it is estimated that 43.2–55.6% of adults with type 2 diabetes do not meet the reference target for glycemic control (hemoglobin A_1c_ [HbA_1c_] <7.0%) [1]. Factors that may contribute to sub-optimal blood glucose (BG) control include inadequate home BG monitoring, non-adherence or non-compliance with medications or lifestyle changes (nutrition and sport), sub-optimal patient education about the disease, and limited access to health professionals [2].

In this context, telemedicine may be an effective approach to solve problems of education, compliance, and monitoring and provider access [3]. BG control could be safely improved by changing the drugs on home BG readings and transmitting them in near-real time to providers. In this setting, telemedicine may also be an effective solution to monitor the complications of the diabetes, especially macro-vascular complications (e.g., myocardial infarction, heart failure) and co-morbidities (e.g., arterial hypertension).

In this article, we review with a pragmatic mind and a clinical vision in the field of remote monitoring (telemonitoring) of diabetic patients.

## Search Strategy

*A literature search has been performed on the PubMed database of* the *US National Library of Medicine* (*https://www.ncbi.nlm.nih.gov/pubmed*) and on *Scholar Google* (*https://scholar.google.fr/*). We searched for articles published between January 1990 and December 2018, using the following key words or associations: “*diabetes mellitus*”, “*telemedicine*” and “*telemedicine in diabetes mellitus*”; restrictions included: language (“*English*” or “*French*”); and type of publication (“*Clinical trials*”, “*Review articles*” and “*Guidelines*”). Textbooks on telemedicine and information gleaned from international meetings were also used.

Our main objective is to provide practical information to clinicians on the benefits of telemonitoring, documented from the current medical literature data. In this context, the selected studies have been classified using the GRADE tool (Grading of Recommendations, Assessment, Development and Evaluations) to indicate the level of evidence (*https://bestpractice.bmj.com/info/us/toolkit/learn-ebm/what-is-grade/*). The GRADE tool proposes an evidence classification ranging from very low to high, as follows: “*Very low = The true effect is probably markedly different from the estimated effect*”; “*Low = The true effect might be markedly different from the estimated effect*”; “*Moderate = The authors believe that the true effect is probably close to the estimated effect*”; “*High = The authors have a lot of confidence that the true effect is similar to the estimated effect*”.

We have reviewed 201 references, which yielded 85 potentially relevant papers. After selection, only 29 papers have been included in our narrative review (defined as a comprehensive, critical and objective analysis of the current knowledge on a topic) and analyzed. Only published telemedicine trials or studies including a clinical evaluation, potentially useful to the clinician in everyday practice have been included in this review. It is to note that the present narrative review is limited by its focus on non-invasive telemonitoring in diabetic patients.

## Telemedicine Studies from 1990 to 2010

**Figure 1:**
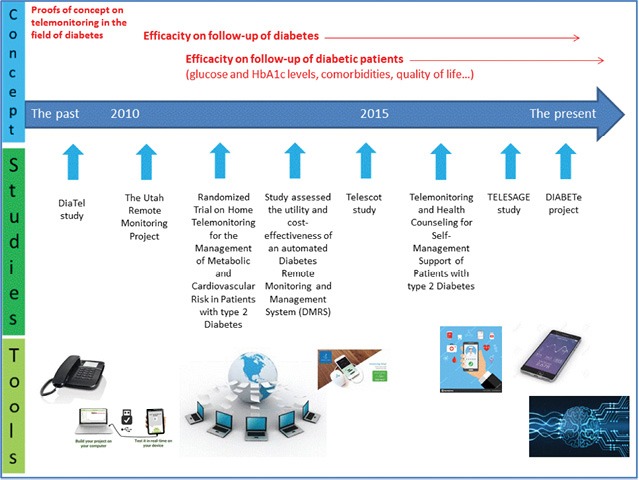
Telemonitoring in diabetes: evolution of concepts and technologies, with a focus on results of the more recent studies.

Since the early 1990s to the end of 2010, more than 20 telemedicine projects and studies have been developed in the field of diabetes and its management [4–27]. Practically all of them have investigated “*structured-telephone support*” (defined as a remote management that can be provided through structured telephone contact between patients and healthcare providers – with or without home visits and reporting of symptoms and/or physiological data) or “*telemonitoring*” (defined as the use of information technology to monitor patients at a distance). These studies have been designed only to monitor BG levels (Figure 1) [4–27]. The first were more like “*proof-of-concept studies*” (defined as a demonstration in principle with the aim of verifying that some concept or theory has practical potential).

All of these studies were GRADE classified as “*Very low*” or “*Low*”.

For the majority of patients, they conducted on:

– Type 1 and type 2 diabetic patients (e.g., children and young people [n=3] or elderly patients [n=2]);
– Patients with intensified therapy (at least two oral anti-diabetic agents + insulin therapy) (n=2) or under insulin pump therapy (n=1);
– Patients with complicated or complex diabetes (e.g., co-morbidities and cardiovascular complications) (n=2).

These studies have involved telemonitoring through the upload and direct transmission of BG data by diabetic patients to providers via cell phone, telephone land line, or Internet-based program [4–27].

The results of these telemedicine projects differed from study to study, with fairly inconclusive results, particularly regarding the statistical significance of the results [3, 28]. In view of their objectives, these studies did not make it possible to conclude on the usefulness of telemedicine in terms of diabetes equilibration and management. One of the explanations for these results is the inclusion of relatively well-balanced diabetic patients. Nevertheless, some studies were particularly promising. This is the case of the DiaTel study [26, 27], GRADE classified as “*Moderate*”.

The DiaTel study compared the short-term efficacy of home telemonitoring coupled with active medication management by a nurse practitioner, with a monthly care coordination telephone call on glycemic control, in veterans with type 2 diabetes [26]. The included patients were taking oral hypoglycemic agents and/or insulin for ≥1 year and had HbA_1c_ ≥7.5%). At enrollment, the patients were randomly assigned to either: active care management (AMC) with home telemonitoring (HT) (ACM + HT group, n=73); and a monthly care coordination telephone call (CC group, n=77) [27]. Both groups received monthly calls for diabetes education and self-management review. ACM + HT group participants transmitted BG level, blood pressure (BP), and weight to a nurse practitioner; the nurse practitioner adjusted medications for glucose, BP, and lipid control based on established *American Diabetes Association* targets. Baseline characteristics of the patients in the DiaTel study were similar in both groups, with mean HbA_1c_: 9.4% in the CC group vs. 9.6% in ACM + HT group [26, 27]. Compared with the CC group, the ACM + HT group demonstrated significantly larger decreases in HbA_1c_ (principal criterion) at 3 months (1.7% vs. 0.7%) and 6 months (1.7% vs. 0.8%; p<0.001 for each), improvement occurring by 3 months.

## Telemedicine Studies from 2010 to 2015

Since the 2010–2015, more numerous mature telemedicine projects and studies have been developed in the setting of diabetes management, especially in the setting of telemonitoring [29–32]. These projects and studies have a main objective to evaluate the use of technology to implement medical and cost-effective healthcare management on a large scale for diabetes management.

All of these studies were GRADE classified as “*Moderate*” [29–31] except the Telescot Diabetes Pragmatic Multicenter Randomized Controlled Trial classified as “*High*” [32].

Compared to the aforementioned projects, most of these have incorporated several new tools or processes for a better management of the diabetic patient, they are as follows:

— Tools or processes for medical education, particularly for patient ownership of the disease, better food hygiene, and increased physical activity;
— Tools for therapeutic and hygiene observance;
— Tool to remote co-morbidities (e.g., arterial hypertension, obesity, dyslipidemia);
— Tools for interaction between the patient and healthcare professionals like telephone support centers, tablets, and Internet-sites, smartphones (Figure 1) [29–32].

The characteristics and the results of the main telemonitoring studies conducted in diabetic patients during this period (2010–2015) are reported in Tables 1 and 2 [29–32]. Analysis of these tables shows that at least three of the studies are positive for improving glycemic control (e.g., BG levels and/or HbA_1c_) and improving co-morbidities (e.g., arterial hypertension). In this setting the Telescot Diabetes Pragmatic Multicenter Randomized Controlled Trial is the most clinically convincing and methodologically sound study [32].

**Table 1:** Characteristics of the telemonitoring studies conducted in the field of diabetes during the period from 2010 to 2015.

Name of the study	Type of the study	Characteristics of the included patients	Type of telemonitoring
The Utah Remote Monitoring Project (n=109) [29]	Non-randomized prospective observational pre- and post-intervention study	Patients with uncontrolled type 2 diabetes and/or arterial hypertension	First arm: – Remote monitoring device: blood glucose level, blood pressure, heart rate and weight. The device was programmed to sound an alarm at a pre-specified patient-referred time to prompt the patient to initiate a telemonitoring session – Patient received a series of education messages, focused on teaching patients about their diseases (diabetes, arterial hypertension) and associated co-morbidities Second arm: – Remote monitoring device: blood glucose level, blood pressure, heart rate and weight and telemonitoring with an interactive voice response (IVR) system – Patient received a call from the telemonitoring IVR service at a pre-specified time. Medical providers were contacted either via a note in the electronic medical record (or immediately if there was a concern, in person or by telephone) if there was an out-of-range value (decided by individual providers or clinics as a value that was high or low)
Randomized Trial on Home Telemonitoring for the Management of Metabolic and Cardiovascular Risk in Patients with type 2 Diabetes (n=302) [30]	Randomized, parallel-group, open-label, multicenter study	Type 2 diabetic patients in general medicine	– Remote monitoring device: blood glucose level, blood pressure, heart rate and weight. The telemonitoring system is associated with remote educational support and feedback to the general practitioner
Study assessed the utility and cost-effectiveness of an automated Diabetes Remote Monitoring and Management System (DMRS) (n=98) [31]	Randomized, controlled study	Patients with uncontrolled diabetes on insulin	– DRMS use text messages or phone calls to remind patients to test their blood glucose and to report results via an automated system. The DRMS made adjustments to insulin dose(s) based on validated algorithms
Telescot Diabetes Pragmatic Multicenter Randomized Controlled Trial (n=321) [32]	Randomized, parallel, investigator-blind controlled trial	Patients with relatively well-controlled type 2 diabetes, with an HbA1c > 7.46%	– Telemonitoring intervention involved self-measurement and transmission to a secure website (weekly twice, morning and evening blood glucose level)

**Table 2:** Results of the telemonitoring studies conducted in the field of diabetes during the period from 2010 to 2015.

Name of the study	Results
The Utah Remote Monitoring Project (n=109) [29]	Principal criteria: – Mean HbA_1c_ had decreased from 9.73% at baseline to 7.81% at the end of the program (p<0.0001) – Systolic blood pressure (BP) had decreased from 130.7 mmHg at baseline to 122.9 mmHg at the end (p=0.0001) Secondary criteria: – Low-density lipoprotein content had decreased from 103.9 mg/dl at baseline to 93.7 mg/dl at the end (p=0.0263) – Knowledge of diabetes and arterial hypertension have increased significantly (p<0.001 for both) – Patient engagement and medication adherence also have improved, but not significantly – Per questionnaires at study end, patients felt the telemonitoring program had been useful
Randomized Trial on Home Telemonitoring for the Management of Metabolic and Cardiovascular Risk in Patients with type 2 Diabetes (n=302) [30]	Principal criteria: – Mean HbA_1c_ difference of 0.33±0.1 (p=0.001) have been observed between the telemonitoring compared and the control group. The proportion of patients reaching the target of HbA_1c_ (HbA_1c_ <7.0%) had been higher in the telemonitoring group than in the control group after 6 months: 33.0% vs. 18.7% (p=0.009) and 12 months: 28.1% vs. 18.5% (p=0.07) – No difference had been registered for body weight, BP, and lipid profile Secondary criteria: – For quality of life (evaluated with the 36-item Short Form health survey), significant differences in favor of the telemonitoring group, as for physical functioning (p=0.01) and mental health (p=0.005) – On an economic level, a lower number of specialist visits was reported in the telemedicine group: incidence rate ratio of 0.72 (95% confidence interval, 0.51–1.01; p=0.06)
Study assessed the utility and cost-effectiveness of an automated Diabetes Remote Monitoring and Management System (DMRS) (n=98) [31]	Principal criteria: – No significant difference for mean HbA_1c_ between the DRMS and control groups at 3 months: 7.60% vs. 8.10% and at 6 months: 8.10% vs. 7.90% (p=ns) Secondary criteria: – Changes from baseline to 6 months have been not statistically significant for self-reported medication adherence – Changes of diabetes-specific quality of life have been not significant registered, except for the Daily Quality of Life-Social/Vocational Concerns subscale score (p=0.04)
Telescot Diabetes Pragmatic Multicenter Randomized Controlled Trial (n=321) [32]	Principal criteria: – The Mean (SD) HbA_1c_ at follow-up was 7.92% in the intervention group vs. 8.36% in the usual care group]. For primary analysis, adjusted mean HbA_1c_ was 0.51% lower (95% CI 0.22% to 0.81%, (principal criterion) (p=0.0007) Secondary criteria: – Adjusted mean ambulatory systolic BP has been 3.06 mmHg lower (95% CI 0.56–5.56 mmHg, p=0.017) and mean ambulatory diastolic BP has been 2.17 mmHg lower (95% CI 0.62–3.72, p=0.006) among people in the intervention group when compared with usual care after adjustment – No significant differences were identified between groups in terms of: weight, treatment pattern, adherence to medication or quality of life – The number of telephone calls was greater between nurses and patients in the intervention compared with control group: rate ratio of 7.50 (95% CI 4.45–12.65, p<0.0001) but no other significant differences between groups in use of health services were identified between groups

The Telescot study is a randomized, parallel, investigator-blind controlled trial with centralized randomization in family practices in four regions of the United Kingdom (Table 1) [32]. This study included 321 patients with relatively well-controlled type 2 diabetes, with an HbA_1c_ >7.46%. In Telescot Diabetes, 160 people were randomized to the intervention group and 161 to the usual care group. The supported telemonitoring intervention involved self-measurement and transmission to a secure website – twice weekly morning and evening glucose – for review by family practice clinicians who were not blinded to allocation group. The control group received usual care, with at least annual review and more frequent reviews for people with poor glycemic or BP control. HbA_1c_ assessed at 9th month was the primary outcome. The mean (SD) HbA_1c_ at follow-up was 7.92% in the intervention group vs. 8.36% in the usual care group (Table 2). For primary analysis, adjusted mean HbA_1c_ was 0.51% lower (95% CI 0.22%–0.81%, p=0.0007). For secondary analyses, adjusted mean ambulatory systolic BP was 3.06 mmHg lower (95% CI 0.56–5.56 mmHg, p=0.017) and mean ambulatory diastolic BP was 2.17 mmHg lower (95% CI 0.62–3.72, p=0.006) among people in the intervention group when compared with usual care after adjustment. No significant differences were identified between groups in weight, treatment pattern, adherence to medication, or quality of life in secondary analyses. During the study, the number of telephone calls was greater between nurses and patients in the intervention compared with the control group: rate ratio of 7.50 (95% CI 4.45–12.65, p<0.0001) but no other significant differences between groups (in use of health services) were identified between groups.

## Telemedicine Studies from 2015 to the Present

Over the last 5 years, “new generation” telemedicine projects and studies have emerged in the setting of type 1 and type 2 diabetes [3, 33–36]. They support automatic transmission and remote interpretation of patients’ data for follow-up and preventive interventions (Figure 1). These new generation telemedicine projects are often known as “*telemedicine 2.0*” projects (also called “*e-Health 2.0*”), they will utilize new *Information and Communication Technologies* (ICT) (defined as the infrastructure and components that enable modern computing) and the *Web 2.0* technologies (defined as a renewal or evolution of these older technologies or of the Internet itself), later being based on old technologies such as HTML) [37].

Most studies have been constructed on various connected tools (*Bluetooth* and/or *Wi-Fi*) for monitoring type 1 and type 2 diabetes and its co-morbidities, such as glucose meters, BP monitors, heart rate monitors, weighing scales, and pulse oximeters [33–36]. Several projects also include continuous glycemic monitoring solution connected tablets and smartphones and often a video-call [3]. Several of these telemedicine projects use machine learning, also called artificial intelligence (AI), in order to be able to:

— Adjust the BG level to the patient’s activity (software Diabeo^TM^) [34, 35];
— Predict patient risks of diabetes decompensation [36]. In the later situation, the cloud-based software aggregates, cleans, and analyzes patient data to allow for identifying patterns that may indicate potential risks and provide predictive insights on healthcare outcomes, as the software MyPredi^TM^ [36, 38].

In fact, several informatics solutions or tools have been developed and used in chronic diseases monitoring (as diabetes), such as *Artificial Neural Networks* (ANN) algorithms, data mining software, ontology [38].

Besides these tools, it must be emphasized that diabetes telemonitoring may use, as for CHF telemonitoring, implantable invasive devices that send either sporadically or continuous data to the receiving physician (automatic telemonitoring) (*outside the scoop of this paper*) [3, 28]. In management of diabetes, implantable telemonitoring devices for multi-parameters including mainly BG-insulin levels monitoring have recently proven to be an effective approach.

All of the studies conducted from 2015 to the present were GRADE classified as “*Moderate*” [33, 39, 40], except the TELESAGE study classified as “*High*” [34, 35].

### Telemonitoring and health counseling for self-management support of patients with type 2 diabetes

**Figure 2:**
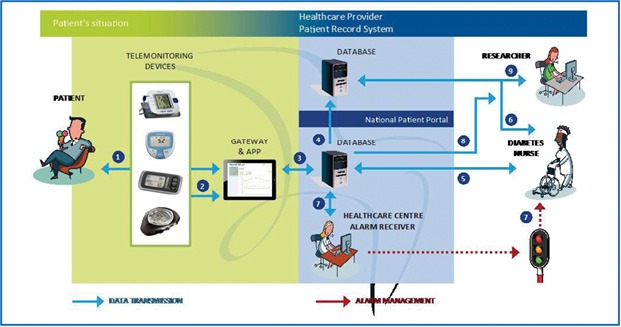
Telemonitoring devices and information flow during the field trial (*adapted from* [33]).

The objective of the Telemonitoring and Health Counseling for Self-Management Support study was to investigate whether the introduction of a health technology-supported self-management program involving telemonitoring and health counseling had beneficial effects on HbA_1c_, other clinical variables (weight, body mass index, BP, blood lipid profile), and *Health-Related Quality of Life* (HRQoL), as measured using the *Short Form Health Survey 36* (SF-36) in patients with type 2 diabetes (Figure 2) [33].

This was a pragmatic randomized controlled trial of patients with type 2 diabetes. Both the control (n=79) and intervention groups (n=87) received usual care [33]. The intervention group also participated in additional health promotion activities with the use of the Prescribed Healthcare *Web* application for self-monitoring of BG and BP. About every second month or when needed, the general practitioner or the DM nurse reviewed the results and the healthcare activity plan. Analysis of the data showed that there were no significant differences between the groups in the primary outcome HbA_1c_ level (p=0.33), and in the secondary outcome HRQoL as measured using SF-36. A total of 80% of the patients in the intervention group at the baseline, and 98% of the responders after 19-month intervention were familiar with using a personal computer (p=0.001). After 19 months, no responders reported significantly poorer mental health in social functioning and emotional role subscales on the SF-36 (p=0.03, and p=0.01, respectively).

### TELESAGE Study

**Figure 3:**
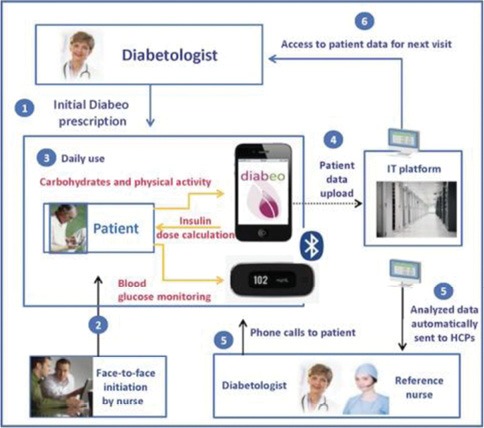
TELESAGE process for diabetic patients assigned to arm 3: (i) Self-measured plasma glucose levels before and after meals (6 measurements) + 1 optional in the night; (ii) carbohydrate counts; and (iii) planned physical activity. HCP: healthcare practitioner (*adapted from* [34]).

TELESAGE (“*Suivi A Grande Echelle d’une population de diabétiques de type 1 et de type 2 sous schéma insulinique basal bolus par la TELEmédecine*”) is a 6-month open-label parallel-group, multicenter study, including adult diabetic patients (n=180), with type 1 diabetes (>1 year), on a basal-bolus insulin regimen (>6 months), with an HbA_1c_ ≥8% [34, 35]. This study will compare a control group (group 1 [G1]: usual follow-up) with two Diabeo^TM^ telemedicine systems: (1) physician-assisted telemedicine (G2), and (2) nurse-assisted telemonitoring and teleconsultations by a diabetologist’s task delegation (G3) (Figure 3).

**Figure 4:**
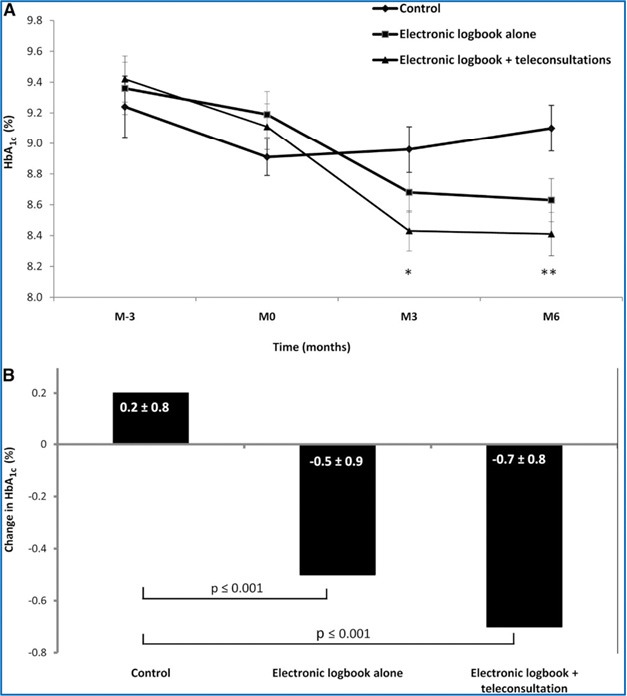
Efficacy of the software Diabeo^TM^, licensed by Sanofi Laboratory. A: HbA_1c_ values (means±SE), from 3 months before baseline to month 6. *p=0.0103, **p=0.0019 compared with control group. B: Change in HbA_1c_ values (means±SE) from baseline to month 6 (*adapted from* [34, 35]).

At 6-month, the mean HbA_1c_ levels were significantly different between the three arms of the TELESAGE study: 8.41±1.04% in G3 vs. 8.63±1.07% in G2 vs. 9.10±1.16% in G1 (p=0.0019 for G1–G3 comparison) (Figure 4) [34, 35]. The Diabeo^TM^ system gave a 0.91% (0.60–1.21) improvement in HbA_1c_ over controls and a 0.67% (0.35–0.99) reduction when used without teleconsultation. There was no difference in the frequency of hypoglycemic episodes or in medical time spent for hospital or telephone consultations. However, patients in G1 and G2 spent nearly 5 h more than G3 patients attending hospital visits.

### DIABETe Project

**Figure 5:**
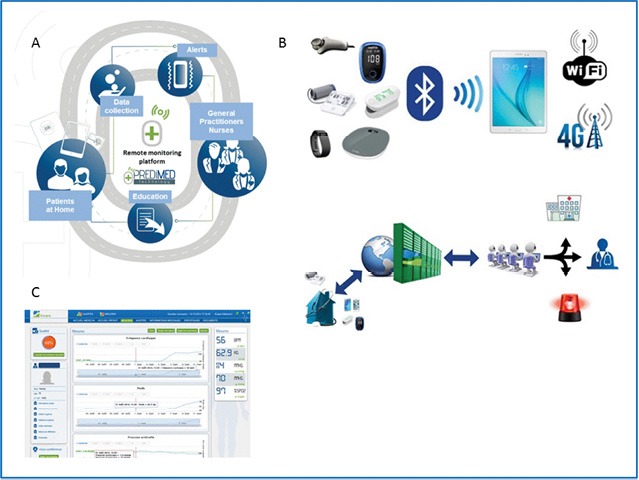
Telemedicine project: DIABETe. A: DIABETe is based on a smart system comprising an inference engine and a medical ontology for personalized synchronous or asynchronous analysis of data specific to each patient and, if necessary, the sending of an artificial intelligence-generated alert (MyPrediTM. B: The platform comprises connected non-intrusive medical sensors, a touchscreen tablet connected by Wi-Fi, and a router or 3G/4G, rendering it possible to interact with the patient and provide education on treatment, diet, and lifestyle. C: The system involves a server that hosts the patient’s data and a secure internet portal to which the patient can be connected to hospital- and non-hospital-based healthcare professionals.

The DIABETe project has been developed to optimize home monitoring of diabetic patients via a telemonitoring 2.0 platform, situations with a risk of decompensation of diabetes and its cardiovascular complications (e.g., myocardial infarction or chronic heart failure [CHF]), the latter ultimately leading to hospitalization [3, 39]. The AI of the DIABETe platform automatically generates indicators of “*health status*” deterioration, i.e., “*warning alerts*” for any chronic disease worsening, particularly diabetes. The platform comprises connected non-intrusive medical sensors (Figure 5), a touchscreen tablet connected by Wi-Fi, and a router or 3G/4G, rendering it possible to interact with the patient and provide education on treatment, diet, and lifestyle. The system involves a server that hosts the patient’s data and a secure Internet portal to which the patient can be connected to hospital- and non-hospital-based healthcare professionals. DIABETe is based on a smart system comprising an inference engine and a medical ontology for personalized synchronous or asynchronous analysis of data specific to each patient and, if necessary, the sending of an AI-generated alert (MyPredi^TM^).

The telemonitoring platform used in DIABETe was first validated in a monocentric study conducted in the Strasbourg University Hospital, carried out as part of the E-Care project, primarily focused on the problem of CHF [39, 40]. One hundred and seventy-five patients (mean age of 72 years) were included into the E-care project, 30% of the patients suffered from type 2 diabetes. During this period, the telemonitoring platform was used on a daily basis by patients and healthcare professionals according to a defined protocol of use which is specific to each patient. During the study, 1500 measurements were taken to generate 700 alerts in 68 patients. One hundred and seven subjects (61.1%) had no alerts upon follow-up. Analysis of the warning alerts in the 68 other patients showed that MyPredi^TM^ detected any worsening of the “patient’s health”, with a sensitivity, specificity, as well as positive and negative predictive values of: 100%, 30%, 89% and 100%, respectively. In this experimentation, both the healthcare professionals and patients, even the frailest, used the E-care system without difficulty until the end of the study.

### Integration of the problems of elderly subjects and co-morbidities associated with diabetes

The challenge for “tomorrow’s” telemedicine is to develop new telemedicine projects and solutions, including the resolution of several medical problems and difficulties, such as [3]:

— The specificities (no appetite for new technologies and uses) and problems (e.g., falls, malnutrition, mild cognitive impairment, etc.) of elderly subjects, who are the main subjects affected by chronic diseases;
— The co-existence of several chronic pathologies (e.g., diabetes, CHF, COPD, etc.) and co-morbidities (e.g., arterial hypertension, renal failure, etc.) in the same individual, while providing comprehensive and “global” care for the individual in all medical and societal dimensions;
— The multiplicity of care structures and medical organizations (e.g., with or without human resources, telemedical centers, etc.);
— The significant logistical barriers to implementing tele-health (Many existing health systems are not designed for these technologies to be integrated within existing information systems.).

In the chronic disease setting, new remote sensors and tailored questionnaires are presently being integrated into the telemedicine platform (e.g., E-care or DIABETEs solutions for our team), including remote actimeters and electronic spirometers, along with new knowledge in the form of ontologies in order to enhance the telemedicine platform and broaden its utility to other chronic diseases like COPD [44, 47].

In this setting, additional personnel and specific protocols are necessary, which must be specific for each chronic disease and targeted for each patient, while allowing the possibility for each patient to exhibit more than one chronic disease. Most of these protocols must still be funded by means of existing resources or external grants.

These diseases share a number of commonalities with diabetes in terms of epidemiology and natural history. Along with diabetes, CHF and COPD are among the most common diseases in developed countries, and thus represent a major public health concern for society [1, 2]. They are accompanied by frequent hospital admissions and re-admissions for well-known causes. These causal factors are detectable, enabling professionals to act ahead of time, as with diabetes and its co-morbidities, thereby avoiding disease progression. Developing warning alerts for these chronic diseases should enhance the existing system.

These points have been addressed by the Whole System Demonstrator cluster randomized trial [41]. In this study, 3230 people with diabetes, COPD, or CHF were recruited from practices by 179 general practices in three areas in England. The patients were divided into 2 groups: usual care or telehealth with remote exchange of data between patients and healthcare professionals as part of patients’ diagnosis and management. In this trial, telehealth has been associated with lower mortality and emergency admission rates. Compared with controls, the intervention group had a lower admission proportion within 12 month follow-up (odds ratio 0.82, 95% confidence interval 0.70–0.97, p=0.017). Mortality at 12 months was also lower for intervention patients than for controls: 4.6% vs. 8.3% (odds ratio 0.54, 0.39–0.75, p<0.001).

Future research must also focus on the accessibility and practicality of telemedicine interventions.

Reimbursement also remains a major concern and a barrier (“glass ceiling”), because much of the care delivered by telehealth is not covered by traditional fee-for-service payment models (e.g., in France, where all HF patients benefit from an integrated processing of healthcare expenses) [28, 36]. The growth of value-based payment models may, however, provide incentives to implement telehealth as a strategy for providing high-quality, cost-effective and coordinated care [36].

At country levels, differences in medical practice laws, restrictions on how telehealth can be delivered, and which patients are to receive these services limits the telemedicine applicability as well.

## Results of Systemic Reviews and Meta-Analysis

To our knowledge, several recent systemic reviews and meta-analysis have been published in the last few months [42–45]. Main results of these works are in accordance with the results of our review. In the work from Wu et al. [42], 19 randomized controlled trials were selected (n = 6294). Telehealth was more effective than usual care in controlling the glycemic index in diabetes patients (weighted mean difference: –0.22%; 95% CI, -0.28–-0.15; p < 0.001). This intervention showed promise in reducing systolic blood pressure levels (p < 0.001) and diastolic blood pressure levels (p <0 .001), while no benefits were observed in the control of body mass index (BMI) (p = 0.79). For total cholesterol and quality of life, telehealth was similar or superior to usual care.

In the review of Hu et al. (14 trials, n=1,324) [44], the use of telemedicine was found to improve HbA_1c_ and reduce the risk of hypoglycemia in diabetic patients, compared to usual care, but without significant difference in BMI. Compared to usual care, telemedicine was found to reduce the odds of hypoglycemia (–0.42; 95% CI = 0.29–0.59; I2 = 32%; p<0.00001). Hu et al. found that the clinical relevance declined in HbA_1c_ level compared to control group (mean difference=–0.28; 95% CI = -0.45–-0.12; p=0.0005), but the telemedicine had no effect on BMI (mean difference = –0.27; 95% CI = –0.86–0.31; p=0.35).

In the review of Lee et al. [45], the use of telemedicine for retinal screening was beneficial and cost-effective for diabetes management with an incremental cost-effectiveness ratio between $113.48/quality-adjusted life year (QALY) and $3,328.46/QALY (adjusted to 2017 inflation rate). Similarly, the use of telemonitoring and telephone reminders was cost-effective in diabetes management.

## Conclusions

This narrative review in the field of telemonitoring with a focus on the more recent studies supports its efficacy in diabetic patients. In this setting, close management of type 1 and type 2 diabetic patients through telemonitoring showed: improvements in control of BG level and significant reduction in HbA_1c_ (e.g., for Telescot et TELESAGE studies [32, 34, 36]); positive impact on co-morbidities (arterial hypertension, weight, dyslipidemia) (e.g., for Telescot and DIABETe studies [32, 39, 40]); better patient’s quality of life (e.g., for DIABETe study) [39, 40]); positive impact on appropriation of the disease by patients and/or greater adherence to therapeutic and hygiene-dietary measures (e.g., The Utah Remote Monitoring Project [29]); and at last, good receptiveness by patients and patient empowerment (Table 3). To date, the magnitude of its effects remains debatable, especially with the variation in patients’ characteristics (e.g., background, ability for self-management, medical condition), samples selection and approach for treatment of control groups.

**Table 3:** Results of the main recent studies on telemonitoring diabetic patients.

	The Utah Remote Monitoring Project [29]	Randomized Trial on Home Telemonitoring for the Management of Metabolic and Cardiovascular Risk in Patients with type 2 Diabetes [30]	Study assessed the utility and cost-effectiveness of an automated Diabetes Remote Monitoring and Management System (DMRS) [31]	Telescot Diabetes Pragmatic Multicenter Randomized Controlled Trial [32]	Telemonitoring and Health Counseling for Self-Management Support study [33]	TELESAGE [34, 35]	DIABETe [39, 40]
Impact on blood-glucose and/or HbA_1c_ levels	+	+	-	+	-	+	•
Impact on arterial hypertension, weight, dyslipidemia and/or other comorbidities	+	•	-	+	-	•	+
Impact on quality of life		+	+	-	-	•	+
Impact on appropriation of the disease by patients and/or greater adherence to therapeutic and hygiene-dietary measures	+	•	-	-	•	•	•
Grade classified	Moderate	Moderate	Moderate	High	Moderate	High	High

“+”: positive impact; “-”: no positive impact; “•”: not studied.

All of these recent studies have been GRADE classified as “*Moderate*” (*the authors believe that the true effect is probably close to the estimated effect*) to “*High*” (*The authors have a lot of confidence that the true effect is similar to the estimated effect*).

To date, relatively few projects and trials in diabetic patients have been run within the “telemedicine 2.0” setting, particularly using AI, ICT and the Web 2.0, as for the studies TELESAGE and DIABETe [34, 35, 39, 40].

Further investigations are needed on efficacy and cost-effectiveness over longer periods of time, and larger samples of diabetic patients.

## Funding

Grants from the *Fondation de l’Avenir*, the *Agence Régionale de Santé du Grand-Est* (ARS) and the *Agence Nationale de la Recherche* (ANR).

## Consent and Ethical approval

Not applicable.

## Competing Interest

M. Hajjam is the scientific director of *Predimed Technology* (www.predimed-technology.fr). All other authors have declared that no competing interests exist.

## Ethical Approval

Not applicable.

## Guarantor

EA.

## Contributorship

EA, LM and MH designed the paper and conducted the literature searches. EA, LM, AAZ and MH drafted the results and parts of the discussion. ST, JD, JH, TB, NJ and AEHH provided critical analysis, revised the whole manuscript, and approved the final version for publication. EA is responsible for all revisions and remains in contact with the rest of the review team regarding status reports.

## Conflict of Interest

The authors confirm that there are no conflicts of interest.
